# Real-time multi-peak tractography for instantaneous connectivity display

**DOI:** 10.3389/fninf.2014.00059

**Published:** 2014-05-30

**Authors:** Maxime Chamberland, Kevin Whittingstall, David Fortin, David Mathieu, Maxime Descoteaux

**Affiliations:** ^1^Centre de Recherche CHUS, University of SherbrookeSherbrooke, QC, Canada; ^2^Department of Nuclear Medecine and Radiobiology, University of SherbrookeSherbrooke, QC, Canada; ^3^Sherbrooke Connectivity Imaging Lab, Computer Science Department, Faculty of Science, University of SherbrookeSherbrooke, QC, Canada; ^4^Department of Diagnostic Radiology, University of SherbrookeSherbrooke, QC, Canada; ^5^Division of Neurosurgery and Neuro-Oncology, Faculty of Medicine and Health Science, University of SherbrookeSherbrooke, QC, Canada

**Keywords:** diffusion MRI, HARDI, tractography, medical visualization, neurosurgical planning, free open source software

## Abstract

The computerized process of reconstructing white matter tracts from diffusion MRI (dMRI) data is often referred to as *tractography*. Tractography is nowadays central in structural connectivity since it is the only non-invasive technique to obtain information about brain wiring. Most publicly available tractography techniques and most studies are based on a *fixed* set of tractography parameters. However, the scale and curvature of fiber bundles can vary from region to region in the brain. Therefore, depending on the area of interest or subject (e.g., healthy control vs. tumor patient), optimal tracking parameters can be dramatically different. As a result, a slight change in tracking parameters may return different connectivity profiles and complicate the interpretation of the results. Having access to tractography parameters can thus be advantageous, as it will help in better isolating those which are sensitive to certain streamline features and potentially converge on optimal settings which are area-specific. In this work, we propose a real-time fiber tracking (RTT) tool which can instantaneously compute and display streamlines. To achieve such real-time performance, we propose a novel evolution equation based on the upsampled principal directions, also called peaks, extracted at each voxel of the dMRI dataset. The technique runs on a single Computer Processing Unit (CPU) without the need for Graphical Unit Processing (GPU) programming. We qualitatively illustrate and quantitatively evaluate our novel multi-peak RTT technique on phantom and human datasets in comparison with the state of the art offline tractography from MRtrix, which is robust to fiber crossings. Finally, we show how our RTT tool facilitates neurosurgical planning and allows one to find fibers that infiltrate tumor areas, otherwise missing when using the standard default tracking parameters.

## 1. Introduction

One of the main features of diffusion MRI consists of the reconstruction of the white matter (WM) fiber pathways in the brain using a computerized technique called *tractography*. The classical way to perform such reconstruction is by following the main diffusion tensor (DT) or orientation distribution function (ODF) direction at each voxel. These techniques fall into the streamline fiber tracking family of techniques (Mori et al., 1999; Tournier et al., 2011). Typically, streamline generation and streamline visualization are done separately. The most common approach is to first reconstruct streamlines using a fixed set of parameters and then visualize the results using one of the different software packages (e.g., MRtrix www.brain.org.au/software/mrtrix, ExploreDTI www.exploredti.com, TrackVis www.trackvis.org, Camino www.camino.org.uk, DTI-Studio www.dtistudio.org, MedINRIA http://www-sop.inria.fr/asclepios/software/MedINRIA, MITK www.mitk.org, BrainVISA www.brainvisa.info, amongst others). However, since calculation and display steps are not linked, a major disadvantage of this approach is that the user cannot directly observe the tracking process. For example, does a slight change in FA threshold dramatically change the appearance and connectivity profile of a certain fiber bundle? Another disadvantage of pre-computing streamline datasets is that parameters used for tractography may not be the same across different subjects and different regions of the same brain (Pierpaoli et al., 1996). This is especially true in pathological processes such as cerebral tumors, lesions or other abnormalities. In a clinical setting, it would be extremely advantageous if neurosurgeons could instantly view how changing tracking parameters impact the results. Lastly, with the ever-growing number of new tracking algorithms, it becomes a real challenge to set these parameters individually to obtain optimal results. One way to visualize the impact of parameters and do some quality assurance (QA) is to link the rendering stage to the tractography algorithm and have a new technique to visualize the uncertainty and validity across parameters.

In this work, we present a real-time fiber tracking (RTT) solution which links the computation and visualization stages in a timely and easy-to-use fashion which permits the user to instantaneously compute and visualize resulting streamlines. Streamlines are directly computed and displayed in real-time whenever a tracking parameter is changed (seeding, masking, step size, maximum angle, weights on input and current directions), or even when seeds are moved in the 3D space. We introduce a fully interactive multi-direction fiber tracking method, which we termed *MultiPeak*-RTT. This new module was added to the FiberNavigator (www.github.com/scilus/fibernavigator) (Vaillancourt et al., 2011). To our knowledge, this is the first comprehensive description of such an algorithm. A diffusion tensor imaging (DTI) RTT module is also supported for groups that only have DT reconstructions. The contributions of this work are four-fold: (1) We propose a novel multi-direction tracking evolution equation that is independent of the underlying field of direction, which, (2) leads to a real-time implementation. (3) We quantitatively validate the MultiPeak algorithm on the *Tractometer* (Côté et al., 2013) and perform a comparison with state-of-the-art offline MRtrix (Tournier et al., 2012) HARDI tracking. (4) Lastly, we show that this MultiPeak-RTT can be used to efficiently visualize important tracking parameters, which we found to vary considerably from area to area in both a healthy control and tumor patient.

## 2. Materials and methods

### 2.1. Datasets

Datasets were collected on a Siemens 1.5 T imaging system using a single-shot echo-planar (EPI) spin echo sequence (TR/TE = 12,500/95 ms), with *b*-value of 1000 s/mm^2^ and 64 uniform directions. Native dimensions were upsampled from 128 × 128 × 60 isotropic 2 mm^3^ voxels to 256 × 256 × 120 isotropic voxels of 1 mm^3^ using trilinear interpolation, as in Dyrby et al. (2011); Raffelt et al. (2012); Smith et al. (2012). In addition, a T1-weighted 1 mm isotropic MPRAGE (TR/TE 6.57/2.52 ms) image was also acquired as an anatomical reference. In this work, Dataset 1 comes from a healthy control volunteer (HC). Dataset 2 consists of a 28-year-old right-handed female patient with left supplementary motor area (SMA) grade III anaplastic astrocytoma, which we refer to tumor patient (TP). Consent was obtained from all subjects. Dataset 3 consists of a revisited version of the FiberCup (FC) (Poupon et al., 2008, 2010; Fillard et al., 2011) with 64 × 64 × 3 isotropic 3 mm^3^ voxels, 64 directions, b = 1500 s/mm^2^. Signal-to-noise (SNR) ratios of the b = 0 image of datasets 1, 2, 3 are 30, 30, and 15, respectively.

### 2.2. Important tractography parameters

Each tractography algorithm comes with its list of parameters that must be adjusted with care, depending on the study or experiment performed. For instance, the parameters used to analyze data from healthy subjects might differ from those in patients with brain tumors, where anisotropy is different and white matter structures may have been perturbed or displaced by the mass-effect of a tumor. Here, we briefly review five main parameters (tracking mask, step size, interpolation, maximum angle, seeding strategy) used in streamline tractography algorithms and discuss how subtle variations can lead to different results.

#### 2.2.1. Tracking mask

Firstly, tractography must be performed on a certain domain and defining a good tracking mask is a crucial step. In most cases, tracking is carried out within a mask defined by a thresholded fractional anisotropy (FA) map, or more recently, generalized FA (GFA) (Tuch, 2004) and apparent fiber density (AFD) (Raffelt et al., 2012; Dell' Acqua et al., 2013) maps. Fiber tracts can be generated by using an permissive thresholds (e.g., FA > 0.1 Castellano et al., 2012) or by using a more restrictive threshold (e.g., FA >0.2). Another way is to define a robust white matter mask using a high resolution anatomical image (T1-weighted image) (Guevara et al., 2011; Girard and Descoteaux, 2012; Smith et al., 2012).

#### 2.2.2. Step size

Secondly, during the tracking procedure, discrete steps, *s*, are taken to track through the white matter. As it stands, there is no consensus on its optimal value even though variations in this parameter can have important effects on the tracking procedure. Too large of a step has the risk of stepping outside a bundle and into another one while a small step size can potentially accumulate numerical errors and increase computational burden.

#### 2.2.3. Interpolation

Thirdly, interpolation is often needed during the tracking process, where the tracking often falls outside the acquired voxel grid. Should interpolation be done on the original DW data, on the field of DTs/ODFs or simply on the principal directions extracted? This remains an open question that has not been thoroughly elucidated in the literature (Pajevic et al., 2002; Dyrby et al., 2011; Raffelt et al., 2012; Smith et al., 2012).

#### 2.2.4. Stopping criteria

Next, another choice to make is the stopping criteria. This highly depends on the fiber tracking algorithm and how it is implemented. In general, the fiber tracking process is always stopped when stepping outside the tracking mask. Moreover, there exists a maximum allowed radius of curvature (*R*) between two consecutive directions or a maximum cone or aperture angle (θ) permitted. The angle used depends on the application and authors in many publications (for review see Descoteaux and Poupon, 2014). Note that the mathematical relationship between *s*, *R* and θ is: θ = min (2 arcsin (*s*/(2R), 90) ∈ [0, 90°], as defined in Tournier et al. (2012).

#### 2.2.5. Seeding strategies

Finally, there exists two seeding strategies: (1) Region-of-interest (ROI) seeding and (2) Complete seeding. In the first case, a ROI is manually or automatically defined from the anatomical reference or an anisotropy measure (often FA), and then, tracking is initiated from voxels contained in this ROI. The second strategy is to track from everywhere in the tracking mask. Depending on the resolution and how many seeds are placed per voxels, this can produce several gigabytes of streamlines. Whole-brain fiber tracking supposes that the tracking parameters are *the same for all* fiber bundles of the brain. Geometry, length, location and pathology can impact the best choice of tractography parameters.

Taken together, the quality and reproducibility of tractography results greatly depends on the chosen parameters. RTT can help in this regard by allowing the user to instantaneously visualize how results change with parameter modification and possibly converge on optimal settings. It can also be of great help to perform fiber generation, exploration and QA.

### 2.3. Existing visualization and RTT literature

Previous studies have investigated the feasibility of visualization based on a set of pre-computed offline streamlines (Peeters et al., 2006; Reina et al., 2006; Petrovic et al., 2007; Hlawitschka et al., 2008; Eichelbaum et al., 2013). These methods focus on how to efficiently render a set of streamlines generated by offline tractography algorithms, for visualization only. On the other hand, real-time processing of DTI tracking methods was achieved by Graphic Processing Units (GPUs) (Jeong et al., 2007; McGraw and Nadar, 2007; Kohn et al., 2009; van Aart et al., 2011). (Mittmann et al., 2011) were the first to present a novel approach in terms of interactivity, which they termed “real-time fiber tracking,” allowing the user to tune a series of tracking parameters “on the fly” and see the instantaneous changes reflected on streamlines. However, this approach is limited to relatively small datasets (i.e., native diffusion dimensions) and is also solely based on tensor fields, which does not take into account the known limitations of DTI in areas of curved and crossings fibers (see Tournier et al., 2011 and references therein). A recent probabilistic method was introduced by Xu et al. (2012), focusing on accelerating Markov-Chain Monte-Carlo (MCMC) tractography methods which are computationally expensive operations. They focused on the GPU implementation, but have not shown that the technique is interactive. Golby et al. (2011) proposed a neurosurgical application using fiducials acting as seeding regions for real-time fiber tracking. The technique is based on DT fields and the number of seed points used must be relatively low to reduce latency. There is therefore a great need in the dMRI community for an integrative software solution that resolves these limitations.

### 2.4. Novel multi-peak real-time tracking algorithm

#### 2.4.1. Implementation details

Our new real-time fiber tracking method is implemented on CPU and runs on a single core computer, which does not require any specific hardware as opposed to CUDA implementations by Mittmann et al. (2011); van Aart et al. (2011); Xu et al. (2012), which are only supported by NVIDIA graphic cards. Computation is done in C++ while the rendering is done with calls to OpenGL and GLSL shaders (Rost, 2006). It is fully open-source and was added as a new module to the FiberNavigator (www.github.com/scilus/fibernavigator) (Vaillancourt et al., 2011).

**Figure 1:**
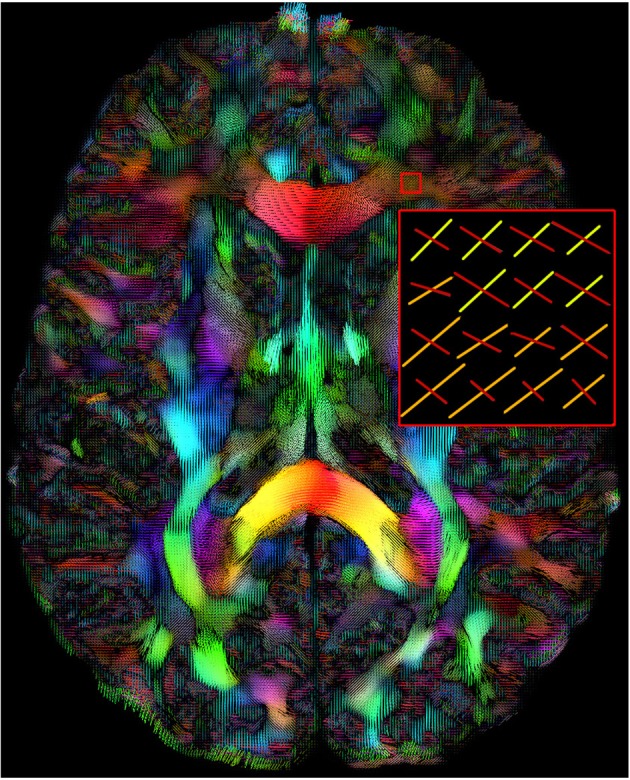
**Maxima map of upsampled fODFs showing multiple directions at each voxels**. The color code is a mapping of their local coordinates (*x*, *y*, *z*) to the red-green-blue channels [R, G, B]. The length of the maxima can also be adjusted interactively by the user. (HC dataset).

The MultiPeak-RTT module is based on a field of upsampled directions as seen in Figure 1. These directions can come from any HARDI reconstruction technique, multi-compartment modeling techniques or model-free techniques (Seunarine and Alexander, 2009; Descoteaux and Poupon, 2014). Here, we use directions that come from maxima extraction on a field of fiber ODFs (Tournier et al., 2007; Descoteaux et al., 2009). We adopted the file format of MRtrix, where the peaks are encoded in a [*X*, *Y*, *Z*, 3*n*], 4D nifti file where *n* represents the number of peaks per voxel. These maxima can be estimated directly with the FiberNavigator or from MRtrix (*find_SH_peaks* command) or any other software.

#### 2.4.2. Tractography algorithm

From these maxima, we have implemented the following streamline tracking algorithm, which can be viewed as a multi-direction vector field extension of TEND (Weinstein et al., 1999; Lazar et al., 2003). The seeds are initiated by randomly choosing a direction from the maxima located at the current voxel, weighted by its norm. A step size, *s*, is then performed in this direction. When entering a new voxel, the peak that forms the smallest angle with the incoming direction **V**_*n* − 1_ is marked as **V**_*n*_ and introduced in the following evolution equation:

(1) $$V_{n\;+\;1}=\left(fV_{n}\right)+(1-f)\left((1-g)V_{n\;-\;1}+gV_{n}\right),$$

where *f* is the FA (or GFA, AFD, or any other underlying scalar map or mask) at the current voxel and *g* is the weight parameter between the in (**V**_*n* − 1_) and out (**V**_*n*_) directions that can be tuned in real-time. This way, streamlines are encouraged to propagate in the current direction when *f* is high and slowed down by their incoming direction otherwise. Note that using a white matter probabilistic map that varies between 0 and 1 is a good choice of *f* map.

#### 2.4.3. Interpolation

As one can see, trilinear interpolation of directions is not computed between steps. This is what makes the method feasible in real-time on CPU. Other classical deterministic methods who perform “offline” tractography must explicitly generate a new local representation (e.g., tensor, ODF, fODF, etc.) after each step by gathering information about the 26 surrounding neighbors. In our case, we accomplish RTT by first upsampling the data, followed by interpolating a new direction on the fly using Equation (1). Previous work (Dyrby et al., 2011; Tournier et al., 2012) mentioned that tracking in an upsampled space (1 × 1 × 1 mm^3^), as opposed to a tracking into diffusion space (2 × 2 × 2 mm^3^), accounts for performing interpolation in the native resolution (diffusion space) on the fiber ODF field. Here, for real-time computation achievements, performing a nearest neighbor interpolation on upsampled data (from 2 × 2 × 2 mm^3^ to 1 × 1 × 1 mm^3^ using trilinear interpolation) will prove to be adequate. Moreover, giving weights to incoming and output vectors also performs some regularization of the streamlines.

### 2.5. User interaction

Our MultiPeak-RTT proposes three interactive tracking methods: a draggable volume of interest (VOI) which acts as a seeding box, a standard mask-based option and a shell-seeding option based on 3D meshes.

**Figure 2:**
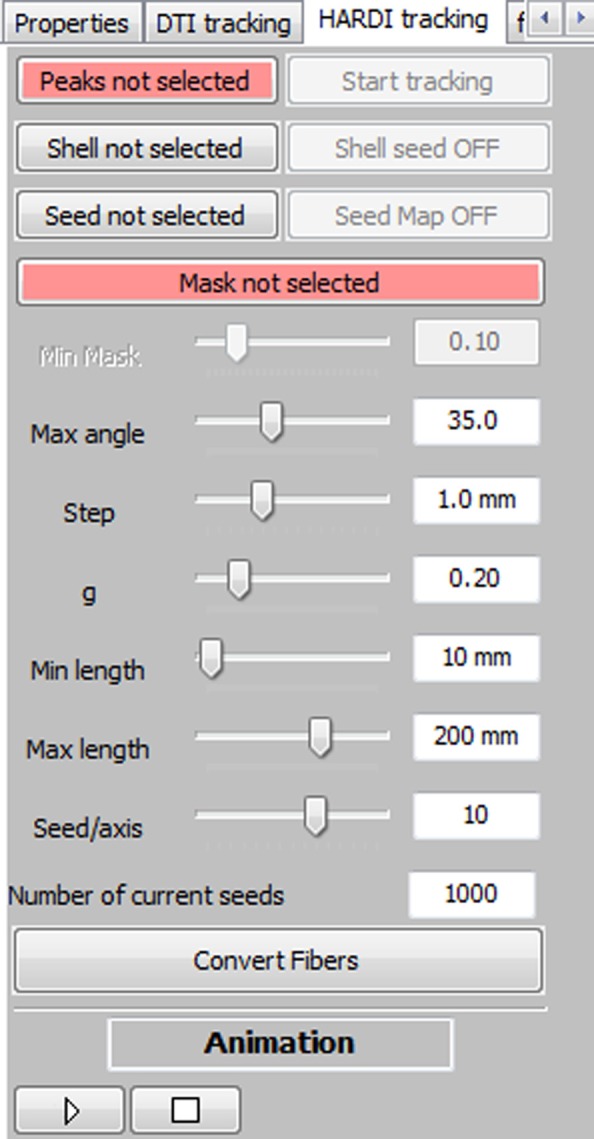
**Overview of the MultiPeak-RTT panel**. The main fiber tracking parameters can be tuned interactively by the user, allowing a more in-depth comprehension of their effects on the resulting streamlines.

The main seeding option consists in a VOI filled with a certain number of seeds that can be adjusted by the user (Mittmann et al., 2011). The box can be moved everywhere within the brain volume and it can be sized to fit the needs of the target region of exploration. The amount of seeds per axis (*x*, *y*, *z*) within the VOI varies between 1 and 15, but the default number of seeds is fixed to 10 × 10 × 10, for a total of 1000 seeds. One can instantaneously generate streamlines while dragging the VOI around. Figure 2 shows the RTT user interface (UI). It includes a series of parameters that the user can modify: the minimum propagation threshold coming from a map (FA, GFA, AFD, or any probabilistic map such as peak intensities or white matter mask), the maximum angle between two consecutive steps, the step size, the weight given to the “in and out” directions (*g* parameter from Equation (1)), the minimum and maximum fiber length, and finally, the number of seeds within the VOI. The basic steps toward interactive fiber tractography are:

1. Specify the diffusion data (i.e., a set of maxima) using the “*Peaks not selected*” button (Figure 2).
2. Provide a diffusion map which will act as a tracking mask (“*Mask not selected*” button).
3. At this step, the user can start the tracking process and interactively explore the data.
4. Finally, if the user wants to save his RTT fibers for further analysis, filtering or save to disk, the button “*Convert Fibers*” will convert the current bundle into a scene object, with editable properties.

The real-time part is really at the heart of this method. The user never has to request streamlines to be updated. By raising a flag each time the seeds are moved in the 3D space and by watching if a parameter has changed, streamlines are automatically recomputed and rendered. This means that there is no queries on pre-calculated streamlines. Each streamline is obtained live.

### 2.6. Visualization techniques

Each seed point spans two streamlines, propagating in both directions of the local corresponding maxima. The basic display of our RTT streamlines consists of rendering those series of points as two arrays of vertices (one containing the front propagation points, the other the back propagated ones) with the use of small line segments. These segments link each point together using GL_LINES, a simple rendering option present in OpenGL. Each part of the streamline is color-coded according to its local direction, which consists in mapping their (*x*, *y*, *z*) normalized coordinates to [R, G, B] values. Whenever the scene becomes passive, the last generated fibers are stored for static rendering, which means that there is no unessential computation performed when the VOI stays still. The array containing the streamlines updates itself whenever the seeds start moving again or a change of parameter is detected. Another rendering technique consists of displaying each point forming the streamline (specifically changing the OpenGL rendering option to GL_POINTS) without linking them together with line segments. This visualization method emphasizes the effect of the step size parameter, allowing close inspection of trajectories points within the 3D environment.

**Figure 3:**
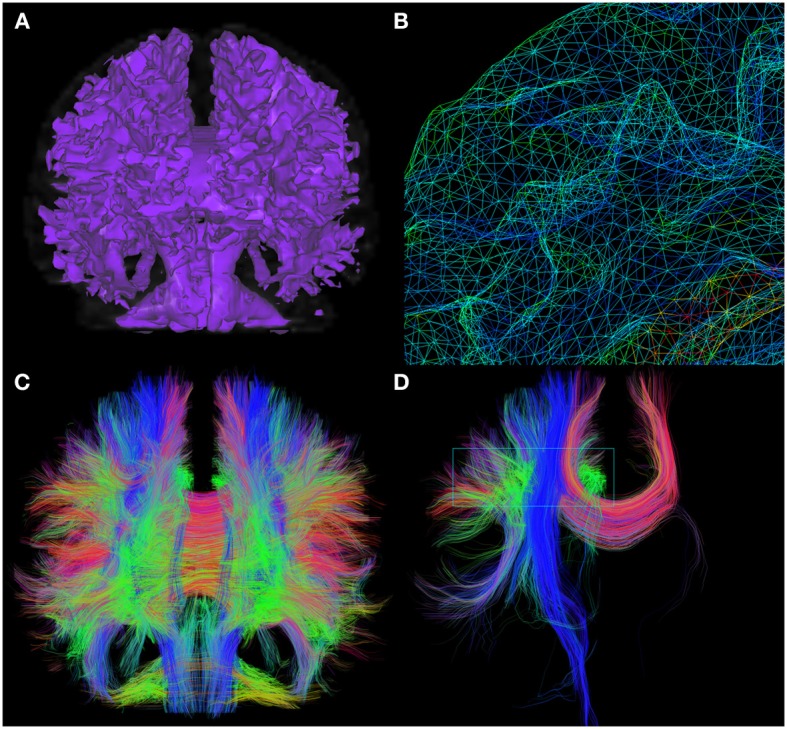
**Whole brain fiber tractography obtained via the shell-seeding option**. In **(A)** an isosurface is generated from an anatomical T1 map to fit the boundaries of GM/WM matter interface. Seed points are then launched at every vertex of the surface **(B)**. **(C)** Shows approximately 200,000 fibers generated with this technique (<10 s). These fibers can then be selected with a VOI for precise exploration as seen in **(D)**. (HC dataset).

Another seeding feature of the interactive fiber tracking UI consists in the use of 3D surfaces that acts as shells for seeding. The first intention of this method is to use a tumor segmentation volume or any other ROI to seed from its surface, allowing streamlines to propagate in both directions of the local corresponding maxima, to see if fibers are infiltrating the region, as in Golby et al. (2011). A good example of the diversity offered by this seeding method is illustrated in Figure 3, where a mesh generated from the white/gray matter interface serves as a shell. Fiber tracking is then initiated from each vertex of the mesh to produce a whole brain tractography output, as illustrated in Figure 3. Note that the term “complete” tractography is also often used in the literature (Descoteaux and Poupon, 2014).

Coupled with the aforementioned techniques, we implemented an animation mode. This new feature enables the possibility to animate fiber tracts growing from a VOI, thus allowing the user to view and analyze the tractography propagation. Based on the principles of a movie player, a “play/pause” button starts the animation or stops the rendering at a certain stage. This visualization method can enhance the perception of the tractography algorithm and its behavior. Supplementary material available online at www.youtube.com/watch?v=Kdwm7cQv5PQ illustrates the new animation mode for the live reconstruction of the cortico-spinal tract (CST), where seeds were launched from a VOI placed in the brainstem.

### 2.7. Evaluation and validation

To assess the validity of the new tracking algorithm, qualitative and quantitative analysis of the streamlines generated by Equation (1) will be performed and detailed in section 3. First, a qualitative evaluation of the MultiPeak-RTT and DTI-RTT was performed. Then, qualitative and quantitative comparisons are done between MRtrix and our MultiPeak-RTT, on phantom and real brain data. It is important to mention that the latter analysis was performed on the upsampled data (1 mm^3^) for both MultiPeak-RTT and MRtrix methods. Finally, to demonstrate the interactive rate of our real-time implementation, we monitored its computational performance.

## 3. Results

### 3.1. Hardi versus DTI

It is well known in the dMRI community that DTI tractography has its limitations (Tournier et al., 2011). Most of the time, DTI methods cannot overcome complex regions with crossing, kissing, and branching since the diffusion tensor becomes isotropic or planar. Hence, in this section, we compare our new MultiPeak-RTT to our previous DTI-RTT (Chamberland et al., 2012) based on the tensor line implementation (Lazar et al., 2003) to ensure that our method can locally resolve those complex crossing regions. The experiment was performed on the healthy control (HC) dataset.

**Figure 4:**
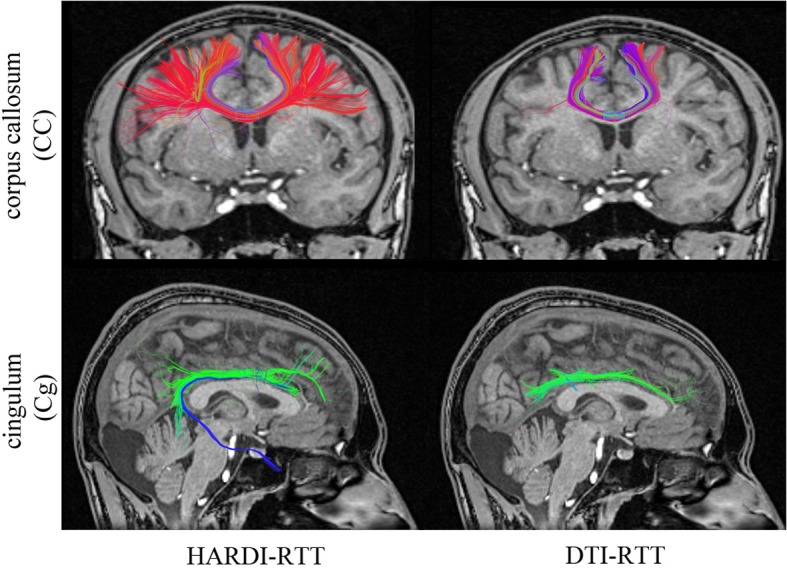
**Streamlines obtained with HARDI (left column) and DTI (right column) tractography**. HARDI reconstructions reveals lateral fibers which are not present in the DTI reconstruction. Bottom row reveals the bending part of the Cg, a part that is often missing when performing DTI-based tractography. (HC dataset).

The main objective was to reconstruct the corpus callosum (CC) and the cingulum (Cg) bundles in their full extent, as best as possible, giving free liberty to the parameters used in both cases. Seeding regions were manually placed in the body of the CC and Cg ( 8 × 8 × 4 mm^3^ and 4 × 4 × 4 mm^3^, respectively). The callosal fibers interconnect homotopic contralateral regions in frontal, central and parietal brain areas. The cingulum is a C-shaped fasciculus running just above the corpus callosum which connects the subcallosal and paraterminal gyri of the frontal lobe with the paracentral lobule, the precuneus and the hippocampus (Catani and Thiebaut de schotten, 2008; Fortin et al., 2012). Table 1 describes the parameters involved in the tracking process for both MultiPeak (e.g., HARDI) and DTI-RTT methods. As one can note, the step size was increased to 1.5 mm for the DTI-RTT CC reconstruction to help the method overcome the crossing regions and find some lateral projections of the CC. The same reasoning was applied for the maximum angle parameter, but this time aimed to catch the lower projections of the Cg to the entorhinal cortex (see Figure 4). The *g*-parameter was also adjusted up to 0.80 so that the newly picked direction was given more weight than the incoming one (see Equation 1). By doing so, streamlines were encouraged to propagate downward the temporal pole instead of aborting their course prematurely. Again, having the ability to see the effect of the step size is a major advantage of performing real-time tractography. The same number of seeds was used in all cases (15 × 15 × 15 = 3375) to enhance visualization and increase the number of streamlines for the CC and Cg reconstructions.

**Table 1:** **Parameters used for HARDI and DTI real-time tractography of the CC and Cg fiber bundles**.

**PARAMETERS**	**HARDI**		**DTI**	
	**CC**	**Cg**	**CC**	**Cg**
Threshold (FA)	0.15	0.10	0.15	0.10
Max angle (θ)	35	35	60	65
Step size (mm)	1	1	1.5	1
*g* parameter	0.25	0.80	0.20	0.25
# of seeds	3375	3375	3375	3375

One can notice the difference in θ and *g* parameters to allow the best reconstruction of these bundles.

The improvement gained from HARDI over DTI is depicted in Figure 4. As expected, the new MultiPeak-RTT method clearly overcomes the DTI limitations by finding more fanning and crossings of the CC projections to the lateral cortices (top-left). It also recovered the lower part of the Cg, illustrated in blue (bottom-left). One can notice that “best” choices of tracking parameters vary depending on the bundle tracked, showing the usefulness of giving access to tractography parameters. It is also important to mention that the reconstruction was instantaneous for both HARDI and DTI tracking algorithms.

### 3.2. Tractometer analysis

**Figure 5:**
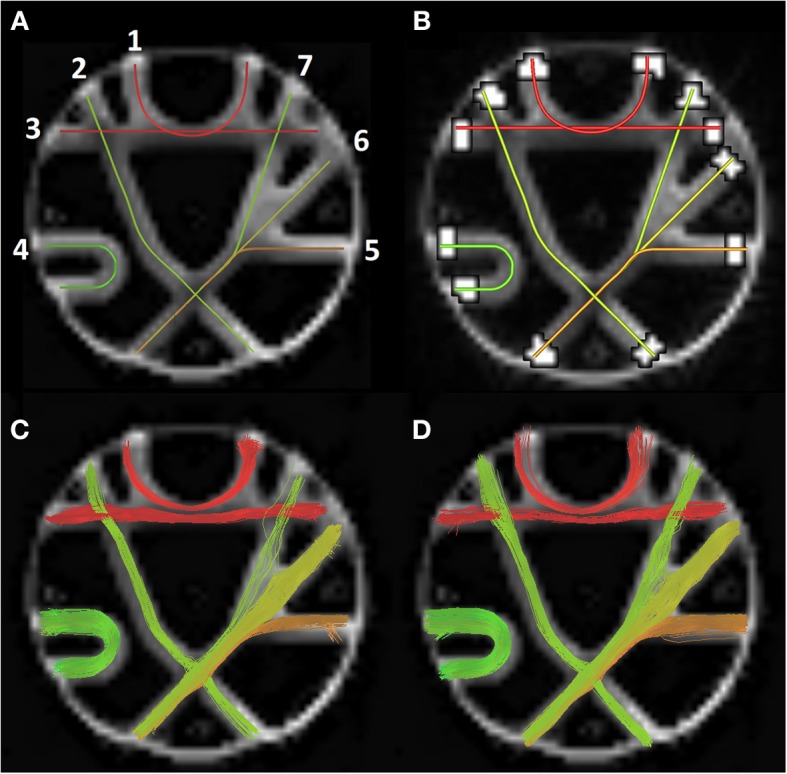
**(A)** The seven FiberCup reference bundles (Poupon et al., 2008; Côté et al., 2013). **(B)** ROIs used for score computation. Bottom row shows valid connections for best MRtrix parameters **(C)** and associate MultiPeak bundles **(D)**.

The Tractometer (www.tractometer.org) is a new online evaluation tool with the purpose of quantifying and highlighting the output of fiber tracking pipelines (Côté et al., 2013). It can evaluate the end effect of fiber tracking at different levels such as the acquisition parameters (*b*-value, number of directions, denoising or not, averaging or not), the local reconstructions (tensor, q-ball, spherical deconvolution), the tractography parameters (masking, seeding, stopping criteria) and the tractography algorithms (deterministic, probabilistic, geodesics, global). It is based on a revisited FiberCup (Poupon et al., 2008, 2010; Fillard et al., 2011) phantom and provides multiple scores generated from streamlines as described in Côté et al. (2013). One of the most important score is the number of valid and invalid bundles (VB/IB). An algorithm should be able to reconstruct at least all of the seven bundles (see Figure 5A) present while keeping a low number of IB. Another good measure is the percentage of valid connections (VC), which indicates the amount of streamlines that actually connect two expected ROIs.

For this validation, multiple fiber tracking parameters combinations were performed on a single fiber ODF (fODF) field (spherical deconvolution (SD) of rank 8 generated with MRtrix). The following tractography parameters were combined for a total of 128 tractography output: Seeding mask (whole WM mask vs ROI masks seen in Figure 5B), # of seeds per voxel (1, 9, 17, 33), step size in mm (0.1, 0.3, 1, 3) and maximum curvature in mm (0.1, 0.3, 0.6, 1). Tractography was performed using the deterministic approach of MRtrix (SD_STREAM option). The same parameter mixture was then used for MultiPeak, in addition to five *g* parameters (0, 0.25, 0.50, 0.75, 1) for a total of 640 combinations. Then, from the results, the best parameters were kept for both MRtrix and MultiPeak methods, which first maximized the VB and then the IB and VC scores. Table 2 shows the two parameter sets (a: MultiPeak, b: MRtrix) used in both methods that led to the best VB, IB and VC results. We also report other measures like the average bundle coverage (ABC) (expressed as a %), which is the average of the number of voxels crossed by streamlines divided by the total number of voxels in the reference bundle. The average region coverage (ARC) is essentially the same as ABC, only applied to the seed regions.

**Table 2:** **Best parameters used for MultiPeak and MRtrix according to VB, IB, and VC scores**.

**BEST PARAMETERS**	**MultiPeak**	**MRtrix**
Seeding mask	ROI	ROI
Number of seeds/voxel	9	17
Step size (mm)	1	3
Curvature (mm)	0.3	0.3
*g* parameter	0.75	–

As one can see in Table 3, both methods were able to reconstruct all of the seven bundles (7/7) with both sets of parameters. Moreover, the percentage of valid connections and the average bundle coverage for RTT is actually better than for MRtrix's *SD_STREAM* but at the cost of slightly more invalid bundles and invalid connections. Figures 5C,D illustrates the VC for both methods according to the second set of parameters. From these results, the main finding is that our MultiPeak can generate similar quality streamlines as the offline MRtrix deterministic approach, while keeping a comparable ratio of valid/invalid connections (see VC/(VC+IC) row of Table 3). To our knowledge, this is the first quantitative comparison between a real-time and offline tractography algorithm.

**Table 3:** **Pair-wise comparison between Tractometer scores for the best set of parameters (issued from Table 2) in both RTT and MRtrix cases**.

**SCORES**	**PARAMETER SETS**	
	**RTT/MRtrix**	**RTT/MRtrix**
VB	7/7	7/7
VC	0.220/0.132	0.211/0.154
IC	0.049/0.034	0.082/0.051
ABC	0.576/0.516	0.631/0.595
ARC	0.643/0.613	0.711/0.723
IB	16/10	19/16
VC/(VC+IC)	0.82/0.79	0.72/0.75

VB, valid bundles; VC, valid connections; ABC, average bundle coverage; ARC, average region coverage; IB, invalid bundles; IC, invalid connections.

### 3.3. Comparison with state of the art MRtrix on real data

Our new MultiPeak-RTT is based on a vector field of directions extracted from fiber ODFs and thus, it is to be compared to tracking based on the full fODF from MRtrix and its deterministic streamline technique (*SD_STREAM* option). The processing of the fODFs and maxima extraction was also performed by MRtrix, offline.

**Figure 6:**
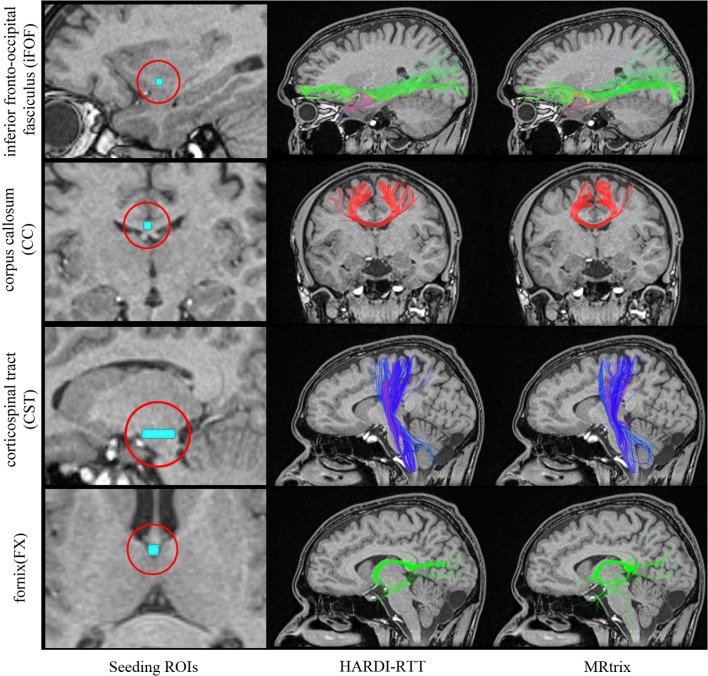
**Qualitative analysis of 1000 streamlines generated from VOIs placed in specific regions of the brain (left column)**. iFOF: VOI was placed in the inferior frontal lobe. CC: ROI located at the middle of the CC. CST: Elongated VOI located at the pontine nuclei level. FX: Seeds where initiated in the middle body of the FX. Center column shows the result of MultiPeak-RTT, while on the right column, the results were obtained using MRtrix (*SD_STREAM*) with default parameters as in Tournier et al. (2012). (HC dataset).

In order to assess the “streamline validity” problem, 1000 tracts were generated from a VOI placed in the same specific regions of the brain for both MRtrix and MultiPeak-RTT, as seen in the first column of Figure 6. The first fiber bundle chosen for this experiment consists in the inferior fronto-occipital fasciculus (iFOF), which connects the infero-lateral and the dorso-lateral frontal cortex with the posterior temporal cortex and the occipital lobe (Catani and Thiebaut de schotten, 2008; Fortin et al., 2012). We also considered the CST, comprising fibers originating from the spinal cord, passing through the pontine nuclei and projecting to the motor cortex. Lateral projections also connects to the motor strip as they cross the centrum semiovale (Catani and Thiebaut de schotten, 2008; Fortin et al., 2012). Next, the fornix (FX) connects the medial temporal lobe to the mammillary bodies and hypothalamus. Its body splits into two branches that runs around the thalamus and connects with the hippocampus (Catani and Thiebaut de schotten, 2008). The last bundle consists in the corpus callosum (CC), which was described in section 3.1.

Since the best tracking parameters to employ varies from an algorithm to another, we simply used the default tracking parameters proposed in both applications (see Table 4).

**Table 4:** **Default tracking parameters used for MultiPeak-RTT and MRtrix**.

**DEFAULT PARAMETERS**	**MultiPeak**	**MRtrix**
Step size	1 voxel	1/10 voxel
Max angle (θ)	35	45
Threshold	0.1 (FA)	0.1 (FOD)
*g* parameter	0.20	–

MRtrix parameters were set according to Tournier et al. (2012).

As one can see on Figure 6, the MultiPeak-RTT algorithm and the MRtrix results are in close qualitative agreement. The reconstructed fiber bundles are similar to those presented in Tournier et al. (2012). The anterior–posterior connections of the iFOF and the highly curved fornix are well represented and almost identical in both cases. On the other hand, the MultiPeak-RTT has slightly more projections in the CC reconstruction than MRtrix and the CST of the MultiPeak-RTT seems to have more coverage of the fanning structure to the motor cortex than MRtrix.

We also quantify the similarity of the MultiPeak-RTT bundles compared to the ones produced by MRtrix. However, in the dMRI community, streamline quantification is still an open problem. There are no consensus on the distance metrics to be used to compare and determine if two fiber bundles are close to one another. We opted for a method used in the image processing and segmentation community, proposed by Descoteaux et al. (2008) based on distance maps generated from a binary segmentation. Here, we first generate a binary map *F*, with 1 if a streamline passes through a voxel. We also allow a tolerance of 1.5 mm as a safe margin of error as proposed in Descoteaux et al. (2008). This is to allow streamlines that run parallel but that are only separated by a voxel to be considered the same. Two different values are then extracted from those maps. Let *a* be the number of voxels that the two datasets have in common, *b* the remaining voxels of the first set *F* of binary voxels that differs from the second set *G* of binary voxels, and *c* the voxels that are present in *G* but not in *F*. The Dice (Dice, 1945) coefficient is defined as the following: κ = 2 | *F* ∩ *G* | /( | *F* | + | *G* | ) = 2*a*/(2*a* + *b* + *c*). The second comparison consists in the degree of overlap between the two set of voxels. Note that this operation can be done in both ways, using MRtrix or MultiPeak-RTT fibers as gold standard since one could map voxels from *F* onto *G* and have a different ratio than mapping *G* onto *F*. It is defined by *r* = *a*/(*a* + *b*). The advantage of using those two metrics is that they range between 0 and 1. Thus, a perfect fit between two bundles will lead to 1, and 0 if there is no overlap at all.

Table 5 shows the ratio of overlap between the MultiPeak-RTT generated streamlines and the ones from MRtrix using the two measures described by the κ and *r* metrics. One can interpret the table as the following: the CC obtained via MultiPeak-RTT accounts for 92% of the voxels obtained from MRtrix's bundle, while in the other way, it covers 93% of the MultiPeak-RTT bundle. Note that all scores range between 0.80 and 0.94 for all fiber bundles which indicates an excellent overlap between the two sets of fibers. The main finding here is that our real-time implementation is equivalent to state of the art offline fiber tractography.

**Table 5:** **Statistical pair-wise comparison between MultiPeak-RTT and MRtrix results using a 1.5 mm overlap tolerance**.

**Bundles**	**Reference**	**Test**	**κ**	***r***
iFOF	MRtrix	RTT	0.89	0.86
	RTT	MRtrix		0.91
CC	MRtrix	RTT	0.92	0.92
	RTT	MRtrix		0.93
CST	MRtrix	RTT	0.92	0.91
	RTT	MRtrix		0.93
FX	MRtrix	RTT	0.86	0.80
	RTT	MRtrix		0.94

### 3.4. Tractography parameter variability across different fiber bundles

**Figure 7:**
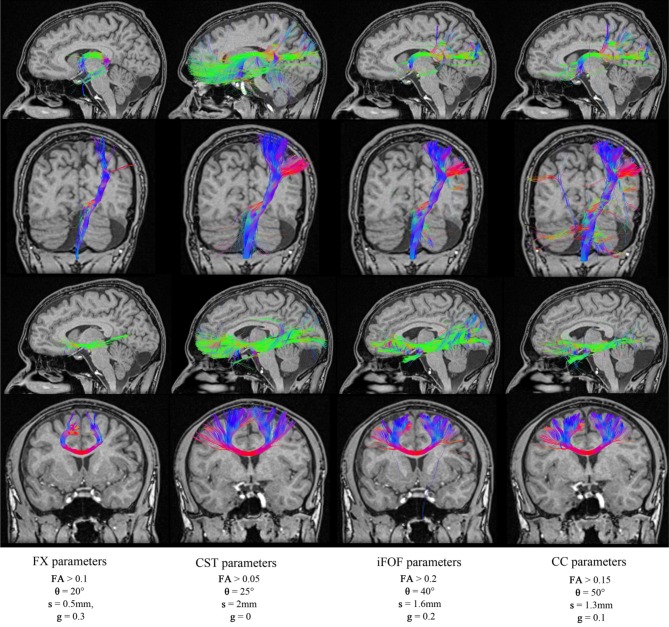
**Reconstructions of four different fiber bundles (FX, CST, iFOF, CC) using a mixture of parameters**. Some of these parameters are best suited for long bundles (CST, iFOF), while others parameters favors the reconstruction of curved fibers. (HC dataset).

To illustrate the variability of tractography parameters across fiber bundles, the best tractography parameters for the reconstruction of the FX, CST, IFOF, and CC were obtained and validated qualitatively by two neurosurgeons (authors David Fortin and David Mathieu). Each of these respective set of parameters are saved (shown in Table 6) and are then re-used in our MultiPeak-RTT to regenerate each fiber bundles with different parameters. As one can see in Figure 7, fixed parameters for the reconstruction of different bundles can lead to misleading and non-optimal results. For example, using the best CC parameters to reconstruct the CST results in undesired streamlines propagating to the right hemisphere (column 4). It is even more crucial when performing fiber tractography on a more complex bundle such as the fornix (Figure 7, first row). It shows multiple false positives streamlines projecting to the undesired cortical areas (columns 2–3) and is also missing the well-known connections (Catani and Thiebaut de schotten, 2008) to the hippocampus (column 4). This phenomenon occurs when the tractography parameters are fixed for a certain bundle, which is not favorable for another bundle with a different shape or location. It is clear that these preliminary results highlight limitations of tractography and how fixing parameters for whole-brain tractography can lead to spurious connections, potentially dangerous for connectomics studies. RTT can help by instantaneously visualizing results and potentially lead to region-based optimization of streamline reconstruction.

**Table 6:** **Best tractography parameters used for the reconstruction of the FX, CST, iFOF, and CC**.

**BEST PARAMETERS**	**FX**	**CST**	**iFOF**	**CC**
Threshold (FA)	0.10	0.05	0.20	0.15
Max angle (θ)	20	25	40	50
Step size (mm)	0.5	2	1.6	1.3
*g* parameter	0.30	0	0.20	0.10

The table shows how optimal parameters can vary across well-known fiber bundles.

### 3.5. Neurosurgical application

**Figure 8:**
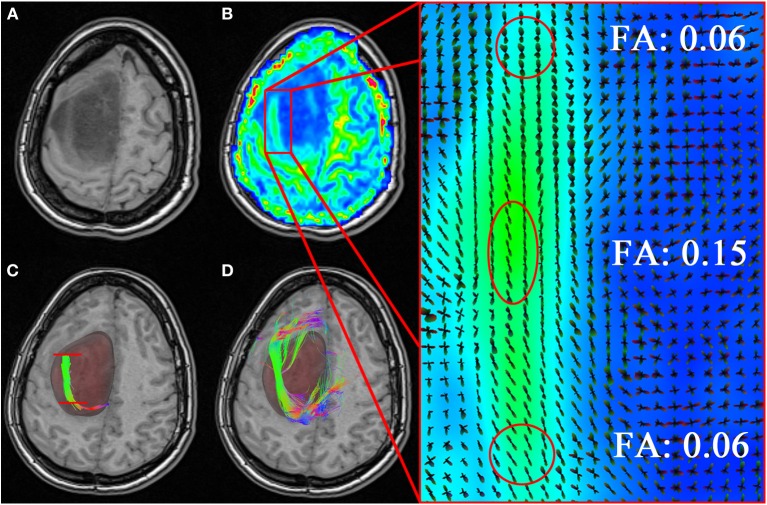
**Neurosurgical application of the MultiPeak-RTT method. (A)** T1-weighted image revealing an anaplastic astrocytoma tumor extending across the left hemisphere of the brain. **(B)** Coherent structure within the tumor region in accordance to local orientations (zoomed rectangle). In **(C)** one can observe a premature termination of the fiber tracts inside the tumor (red lines). By lowering the FA threshold to 0.06, we allow the tracts to momentarily step over small local changes in the diffusivity and continue their course toward the cortex **(D)**. (TP dataset).

Finally, we show how our MultiPeak-RTT can perform in a neurosurgical application by showing the benefits of having the ability to adjust the FA stopping criteria in real-time, and thus, revealing important information otherwise hidden to the surgeon. Figure 8, illustrates the case of a tumor patient (TP) dataset with left SMA grade III anaplastic astrocytoma (see section 2.1). Partial convulsions were signaled as pre-operative symptoms. The patient had no neurological deficits before undergoing surgery. The MultiPeak-RTT permitted to explore the tumor prior to surgery, showing not only relevant streamlines around the tumor, but also infiltrating it. Those streamlines were revealed by reducing the FA threshold within the affected area, thus enabling streamlines to continue their path instead of stopping prematurely. A T1-weighted image showing the tumor spread across the left hemisphere of the brain is shown in Figure 8A. Close inspection of the FA map in **(B)** reveals evident structure within the tumor region (zoomed version of the red rectangle shows coherent ODFs along that structure). According to Castellano et al. (2012), the standard FA threshold is usually at 0.1. In our study, if fiber tracking had been performed offline using an FA threshold of 0.1, relevant fibers surrounding the tumor would not be visible. In fact, with a FA threshold of 0.1, streamlines propagate until they reach a certain barrier caused by small local changes in the diffusivity inside the tumor. The result is a blockage in the tracking algorithm, which is illustrated in **(C)** (red lines). A surgeon may interpret this result as irrelevant since none of the fibers actually connects with regions outside of the tumor. By lowering the FA threshold to 0.06 in real-time, a “temporary bridge” is created, thus allowing streamlines to connect regions of the cortex together **(D)**. This re-enforces the notion that FA thresholds can be highly variable and should be adjusted accordingly in the presence of the neurosurgeon for optimal surgical planning.

This case study shows once again the importance of interactive parameters adjustment for a neurosurgical application. Indeed, more quantitative and qualitative analysis should be performed in the future to determine how MultiPeak-RTT affects tumor resection. Recent work already investigated how fiber tracking can be employed and interpreted in a neurosurgical application (Klein et al., 2010; Golby et al., 2011; Barajas et al., 2013; Kuhnt et al., 2013).

### 3.6. Performance

In order to appreciate a real-time application, the feedback sent to the user must be done at an acceptable rate. In our case, this statement implies that the user should be able to drag the VOI everywhere in the volume and instantaneously see the corresponding streamlines to be displayed on the screen. Mittmann et al., 2011 established a benchmark for performance measurement using the frame per second (FPS) index. In order to be acceptable, they stated that the mean FPS rate of a real-time fiber tracking tool must be greater than 10. To do so, their strategy consists in moving a VOI inside the volume in small steps along the *z* direction, to mimic the user interaction. The VOI has the same shape as the original volume, but resized by a factor ratio of 0.1. Streamlines are then computed and shown at the same time for each iteration of the process. To calculate the FPS ratio, the number of frames it took for the VOI to cover the *z* axis is divided by the time spent doing so. As expected, the FPS rate varies depending on the parameters used in the tractography process. For example, initiating a VOI filled with 15 seeds per axis (for a total of 3375 seeds), with a low FA threshold acting as a tracking mask and a small step size surely results in rendering more streamlines, and thus sending more points to the graphics card. Hence, the experimentation proposed was performed with the default tracking parameters as seen in Figure 2. In order to demonstrate how the seed per axis parameter impacts on interactivity, we tested the sweeping method for 11 different number of seeds, and repeated the process eight times using the HC dataset. Experimentation was done on a laptop with the following system: Ubuntu, Kernel: Linux 2.6.32, Mode: 32-bit, Video card: Geforce GT 435M memory 2048 MB 800 MHz, NVIDIA Driver: 304.43, CPU: Intel(R)Core(TM) i7 Q840 @ 1,87 GHz, 8 GB RAM.

**Figure 9:**
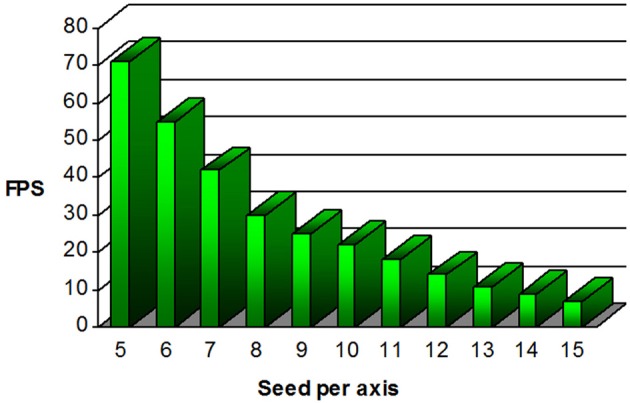
**Graphical representation of the frame per second (FPS) performance regarding the number of seed per axis present within the VOI**. For the default 1000 seeds proposed (10 × 10 × 10), the mean FPS value is over 20.

Figure 9 shows the mean FPS attained depending on the number of seeds per axis used. Recall that fiber tracking and rendering are solely done on a single CPU thread. As one can note, our MultiPeak-RTT implementation respects the minimum acceptable FPS rate of 10 with default parameters. This was further tested in the operating room by two neurosurgeons (authors David Fortin and David Mathieu) who found no issues with software performance and interactivity. It is important to mention that both MultiPeak and DTI-RTT implementations achieved similar performance.

Real-time fiber tractography is heavily dependent on the time spent computing streamlines and more precisely, on the interpolation step. Our method works on a single CPU while maintaining an acceptable FPS rate mainly because the trilinear interpolation is computed only once on the original dataset and not performed during the tracking process. As long as the suggested parameters do not exceed a certain limit, the mean FPS stays acceptable. In order to maintain good performance, our results suggests to maintain seeding under 13 seeds per axis (2197 seeds) while moving the VOI. Then, if needed, one can simply increase that number of seeds when done moving the VOI. This way, a higher number of seeds is instantly generated and rendered into a static scene, for a better coverage of the current region of investigation.

## 4. Discussion

In general, tractography parameters tend to be fixed and repeated from previous experiments. For example, optimal parameters for one specific bundle are not necessarily ideal for another fiber bundle located in a different region of the brain (Figure 7). Allowing the user to instantaneously visualize how results change with parameter choice may lead to optimal settings and facilitate interpretation of tractography results. In this study, we present a real-time multi-peaks fiber tracking method which can facilitate this (www.youtube.com/watch?v=Kdwm7cQv5PQ).

### 4.1. Novel tractography algorithm

A real-time tractography algorithm with a new evolution equation (Equation 1) was introduced in this article. It was validated and compared on phantom and real data against the state of the art MRtrix tractography based on the full fiber ODF. Two parameters came into play, the *f* and *g* parameters, similar to the TEND algorithm (Weinstein et al., 1999; Lazar et al., 2003).

#### 4.1.1. g-parameter

It was observed that using a *g* value between [0.6, 1] often led to noisy streamlines, since the **V**_*n*_ of Equation (1) direction is given too much weight. This makes streamlines step from voxel to voxel without deviation, increasing the chance of hitting the stopping mask. However, resulting streamlines tend to be more constrained, which is good when looking for smaller connections in the brain. For example, if a highly curved bundle needs to be reconstructed (e.g., Cg), we observed that using a *g* value near 1 is better because forgetting the previous direction and emphasizing on the next, which allows easier high curvature turns. At the opposite, giving more weight to the **V**_*n* − 1_ direction by using a *g* value between [0, 0.6] smooths the path, resulting in stepping out of the tracking mask less often. Lower *g* values thus helps in the reconstruction of relatively straight bundles (e.g., CST, iFOF).

#### 4.1.2. f-parameter

On the other hand, the *f* parameter can also be changed, but we preferred to have a dynamic value that varies with the underlying map at each point of the brain. Other DTI methods (Westin et al., 1997; Kindlmann and Weinstein, 1999; Weinstein et al., 1999; Alexander et al., 2000; Lazar et al., 2003) proposed the use of *f* = *cl*, where *cl* is a geometric anisotropy metric for tensor shape. The use of other metrics such as fODFs amplitude (Tournier et al., 2012) or white matter probabilistic masks (Girard and Descoteaux, 2012; Smith et al., 2012) could be used. Overall, both *f* and *g* parameters contribute to regularize the streamline propagation.

### 4.2. Hardi versus DTI

As expected, when compared to the DTI-RTT, MultiPeak-RTT provided better robustness to multiple fiber crossing configurations as well as high curvature regions as denoted in section 3.1. Recent studies have shown that even if DTI-based methods are the most widely spread clinical tractography methods, they can lead to misleading information about fiber tracts orientation (Farquharson et al., 2013). Thus, high-order reconstruction methods should be favored, even from limited DTI-like acquisitions (Girard et al., 2012; Tournier et al., 2013). Our software provides the choice of choosing between single (DTI) or multi directions (HARDI used here) tracking, in a computionnally efficient way.

### 4.3. Comparison with state of the art MRtrix using the tractometer and real data

The quantitative and qualitative comparison with MRtrix showed that the MultiPeak algorithm presented here can compare with state of the art offline techniques, as shown in sections 3.2, 3.3. The use of the Tractometer online evaluation system also permitted to quantify the differences between the two methods (seen in section 3.2).

### 4.4. Neurosurgical application

The MultiPeak-RTT technique was shown useful in a clinical application and is a quick and easy way to optimize tractography in a neurosurgical planing context (see section 3.5). With the use of MultiPeak-RTT, surgery can be individually adapted to the neuro-anatomy of each patient. In our case, using the MultiPeak-RTT results, the extent of resection was tailored to preserve the tract that was shown going through the supero-lateral edge of the tumor (Figure 8D). The patient recovered uneventfully from the surgery, without new neurological deficit. Also, it is no longer necessary to bring several gigabytes of data to the surgery room, since those can now be computed live during the intervention. For example, in the past, we used to bring to the surgery room multiple sets of streamlines to show potential uncertainty and limitations of tractography parameters to the neurosurgeon. Overall, this RTT module opens perspectives for QA prior neurosurgical interventions and faster data generation.

### 4.5. Future work

GPU implementation will surely be considered in the future but we think that real-time probabilistic tracking could also be implemented solely on CPU using similar simplifications as done in this work. In fact, an extension to the current MultiPeak-RTT is to add the uncertainty with respect to each direction coming from prior local modeling computation to be included in a probabilistic MultiPeak-RTT module. Moreover, in the future, we will create an interface for others to add their tracking algorithm in our MultiPeak-RTT framework.

## 5. Conclusion

We presented a new interactive real-time fiber tracking feature that takes into account crossing information issued from any local model able to estimate multiple directions. It overcomes the well-known DTI limitations, while remaining interactive in a real-time adjustable application due to its implementation. We introduced a novel interactive seeding strategy based on 3D surfaces. We also showed that the generated streamlines were in close agreement with ones generated offline by MRtrix tractography. The application gives quick convincing results on the fly and is an important tool to explore specific regions of the brain to find appropriate tractography parameters, given a certain hypothesis, task or application. It permits close inspection of DWI data by enabling the instantaneous display of fiber tracts. This feature allows the user to see the effect of each parameter involved into the tractography process. The tractography algorithm will soon be released in Dipy (Garyfallidis et al., 2014) and is currently open-source in the FiberNavigator (www.github.com/scilus/fibernavigator).

### Conflict of interest statement

The authors declare that the research was conducted in the absence of any commercial or financial relationships that could be construed as a potential conflict of interest.

## Supplementary material

Supplementary video data can be found online at www.youtube.com/watch?v=Kdwm7cQv5PQ.
